# Patterns of fish and whale consumption in relation to methylmercury in hair among residents of Western Canadian Arctic communities

**DOI:** 10.1186/s12889-020-09133-2

**Published:** 2020-07-06

**Authors:** Emily V. Walker, Yan Yuan, Safwat Girgis, Karen J. Goodman

**Affiliations:** 1 Faculty of Medicine and Dentistry, University of Alberta, Edmonton, Canada; 2 School of Public Health, University of Alberta, Edmonton, Alberta Canada; 3 Department of Laboratory Medicine and Pathology, University of Alberta, Edmonton, Canada

**Keywords:** Methylmercury, Fish consumption, Whale consumption, Canadian Arctic, Hair analysis

## Abstract

**Background:**

Methylmercury contamination of the environment represents a substantial environmental health concern. Human exposure to methylmercury occurs primarily through consumption of fish and marine mammals. Heavily exposed subgroups include sport or subsistence fishers residing in Arctic communities. We aimed to estimate the association of fish/whale consumption patterns of Canadian Arctic subsistence fishers with the internal dose of methylmercury as measured in hair.

**Methods:**

This research was conducted within ongoing community projects led by the CAN*Help* Working Group in Aklavik and Fort McPherson, Northwest Territories and Old Crow, Yukon. We interviewed each participant using a fish-focused food-frequency questionnaire during September–November 2016 and collected hair samples concurrently. Methylmercury was measured in the full-length of each hair sample using gas chromatography inductively-coupled plasma-mass spectrometry. Multivariable linear regression estimated beta-coefficients and 95% confidence intervals (CIs) for the effect of fish/whale consumption on hair-methylmercury concentrations.

**Results:**

Among 101 participants who provided hair samples and diet data, the mean number of fish/whale species eaten was 3.5 (SD:1.9). The mean hair-methylmercury concentration was 0.60 μg/g (SD:0.47). Fish/whale consumption was positively associated with hair-methylmercury concentration, after adjusting for sex, hair length and use of permanent hair treatments. Hair-methylmercury concentrations among participants who consumed the most fish/whale in each season ranged from 0.30–0.50 μg/g higher than those who consumed < 1 meal/week.

**Conclusions:**

In this population of Canadian Arctic subsistence fishers, hair-methylmercury concentration increased with fish/whale consumption, but the maximum concentrations were below Health Canada’s 6.0 μg/g threshold for safe exposure.

## Background

Mercury is a chemical element with the capacity to induce potent toxic effects in humans. Because of this, contamination of the environment with mercury compounds represents a substantial environmental health concern. For this reason, mercury has been the focus of a large body of research aimed at identifying the mechanisms through which it enters the environment, as well as pathways for human exposure and subsequent toxicological effects.

When elemental mercury finds its way into aquatic systems, some of it transforms into methylmercury (MeHg) [1–5]. As an organic compound, MeHg is lipophilic and mobile, with the capacity to enter the plasma membrane of cells and accumulate in the cytoplasm [1, 5]. This property has important implications for bioconcentration of MeHg in aquatic organisms and subsequent biomagnification in aquatic food chains [1–5]. MeHg contamination of aquatic ecosystems is considered the most abundant non-occupational source of human exposure to mercury [1–4, 6, 7]. The primary source of MeHg exposure in humans is consumption of fish or fish products and marine mammals, with larger, longer-living fish posing greater risk of exposure [1–4, 6, 7]. The most heavily exposed human population groups include sport or subsistence fishers residing in Arctic communities [1–3, 8]. Arctic fish pose a disproportional threat due to greater emissions of elemental mercury in the northern hemisphere, changes to the global climate altering the mercury cycle, and more frequent consumption of species vulnerable to high levels of organic mercury contamination [1–3, 8]. Additionally, sport and subsistence fishers do not benefit from regulatory measures that control the mercury content of commercially sold fish products.

Research on mercury exposure among Indigenous residents of the Canadian Arctic has typically focused on coastal populations that consume large amounts of marine mammals [9]. This is reasonable, given the greater capacity of these large species to accumulate mercury. Residents of inland communities in the western Canadian Arctic, however, are target audiences for public health messages about fish consumption, without concurrent exposure assessments [10, 11]. Our preliminary ethnographic research in western Canadian Arctic communities revealed residents’ concerns about mercury accumulation in their bodies and how it relates to their fish and marine mammal consumption. In response, we conducted this research to analyze data collected from residents of inland communities in the Canadian Arctic to: characterize fish and marine mammal consumption patterns; biochemically measure the mercury level in hair samples to ascertain individual exposure to mercury; and estimate the effect of fish and marine mammal consumption, other dietary components and participant characteristics on the internal dose of mercury.

## Methods

### Study design

This mercury exposure project was an environmental health component of ongoing community-driven projects led by the Canadian North *Helicobacter pylori* (CAN*Help*) Working Group in western Canadian Arctic communities (www.canhelpworkinggroup.ca). The CAN*Help* Working Group formed during 2006–2008 in response to concerns raised by community leaders about *H. pylori* infection and gastric cancer risk. This research program is a collaborative effort, linking northern Canadian Indigenous communities, their health care providers and regional health authorities with investigators from multiple disciplines at the University of Alberta [12, 13]. At the invitation of community leaders, cross-sectional projects were established to describe the community health burden from *H. pylori* infection and associated disease and address community concerns. In each community, a planning committee made up of community members guided the conduct of each project and ensured that research activities were culturally appropriate and in keeping with community priorities.

### Person, place and time

The mercury exposure project was conducted within three CAN*Help* Working Group community projects. The first of these projects launched in 2007 in the hamlet of Aklavik, Northwest Territories (NT) (2006 census population = 590, ~ 92% identifying as Gwich’in [Athabascan First Nation] or Inuvialuit [Inuit]) [14, 15]. Projects began in 2010 in Old Crow, Yukon (YT) (2011 census populatio*n* = 245, ~ 85% identifying as Vuntut Gwich’in [16, 17], and in 2012 in Fort McPherson, NT (2011 census population = 844, ~ 90% Tetlit Gwich’in) [18]. Participation in the mercury exposure project was open to all residents of these three communities during September–November 2016. Recruitment activities involved radio announcements, social media posts, flyers on community message boards, and directly contacting participants of CAN*Help* Working Group projects for which current contact information was available.

### Informed consent

All participants received an information sheet that outlined the study objectives, methods, information to be collected, benefits and potential risks of participation, and confidentiality protection measures. Following review of this document, each consenting participant filled out a consent form, confirming they had received enough information about the project and agreed to participate. Project information sheets and consent forms were reviewed and approved by the Research Ethics Board at the University of Alberta and have been published previously [19].

### Choice of tissue for biomarker analysis

Evidence suggests that hair is the biological medium best suited for measuring MeHg exposure [1, 7, 20–32]. Hair from the scalp is a commonly selected matrix for biomonitoring of MeHg exposure, because MeHg accounts for approximately 80% of the total mercury found in hair and can be measured directly [1, 7, 20–32]. Practical advantages to collecting hair samples relative to urine and blood include: chemical stability; simple and non-invasive sampling; ease in storing, transporting and archiving specimens; and relatively low cost [21, 33–36].

### Exposure time window

Among healthy individuals, estimates of the scalp hair growth rate range from 0.6 to 3.36 cm/month, with an average of 1 cm/month [33–35]. The concentration of mercury measured in hair reflects exposure over the growth period of the sampled hair, which depends on hair length. According to input from local planning committees, residents of participating communities consume the greatest amount of aquatic species, on average, during the spring and summer seasons. For this reason, hair sample collection took place during the fall season (September–November).

### Hair sample collection

Procedures for collecting hair samples were adapted from protocols outlined by the United States Centers for Disease Control (CDC) for use in the National Health and Nutrition Examination Survey (NHANES) [37]. We collected all hair samples from the occipital region of the scalp using stainless steel shears, obtaining a minimum of 120 mg of hair from each participant to allow for duplication of the laboratory measurements for quality assurance/quality control (QA/QC) purposes. Given that hair length determines the exposure period represented in the strand, we also used a ruler to measure hair length (in cm) before transferring samples into a zip-closable plastic bag and applying a label specifying the sample ID number, collection date, sample weight and hair length. Additionally, we recorded information on use of permanent hair treatments, including hair dye or permanent waves, and time since the most recent treatment.

### Laboratory analysis of samples

The collected hair samples were analyzed by the University of Alberta Biogeochemical Analytical Service Laboratory (BASL). This lab has been accredited by the Canadian Association for Laboratory Accreditation (CALA) as meeting ISO/IEC 17025 standards for the performance of specific tests. MeHg was measured in the full-length of each hair sample using gas chromatography inductively coupled plasma-mass spectrometry (GC-ICP-MS) [38, 39]. Quality control methods employed by the lab included the use of reference material 1AEA-085 for MeHg, total mercury and other trace elements in hair. Single point calibration was applied, and the calibration standard was analyzed in 4 replicates. The relative standard deviation for the ratio of Hg isotope 201:202 was considered acceptable if the value was less than 5%. If the value was greater than 5%, the calibration was repeated. Instrument and method blanks and a second source reference material were also used to monitor contamination with MeHg, accuracy and instrumental drift during analysis. These were incorporated into the analysis at a frequency of 1 per batch of approximately 30 samples. The instrument was re-calibrated if the second source reference material measurements were outside of the 80–120% recovery range. Additionally, water samples were spiked with a known quantity of enriched MeHg isotope (CH_3_^201^Hg) as an internal standard. Finally, laboratory duplicates were performed at a frequency of 1 per 5 samples. For added quality assurance, we divided approximately 10% of the samples into duplicates and submitted them to the lab as separate individuals. Lab personnel were blinded to all participant characteristics, including age, sex and the amount and types of aquatic species consumed.

### Fish and marine mammal consumption data

We designed a population-appropriate Food Frequency Questionnaire (FFQ) focused on fish and marine mammal consumption in the past year for this study (Supplemental File 1). Community input guided the selection of included fish species and incorporation of familiar names and descriptions for locally harvested fish, to ensure respondents had a clear understanding of each FFQ item. Planning committee members identified Beluga Whale (*D. leucas*) as the only regularly consumed marine mammal. The FFQ measured consumption frequencies as average number of times each type of fish or whale was consumed per week (we will refer to food consumption events as “meals”). The FFQ did not include portion size to reduce the burden on participants and because validation studies have shown that attempting to ascertain portion size does not appreciably improve overall characterization of diet, because most people have poor recall of portion sizes [40]. To capture seasonal variability in consumption, the FFQ asked respondents to specify the time of year in which they typically harvest each aquatic species they reported consuming. The FFQ then asked respondents the typical number of meals per week of each species during the time of year they are harvested. Since it is common for community members to preserve harvested fish by drying, freezing or smoking the meat, the FFQ asked respondents to report the frequency of consuming each species during other parts of the year. Given the potential for preparation methods to alter the bioavailability of mercury in consumed fish, the FFQ also asked participants to specify how they typically prepared each type of fish/whale for eating and the parts they consumed [41]. Most participants were able to identify the specific species they consumed; pictures were available for those who were unsure. The potential for the overall composition of an individual’s diet and intake of specific nutrients to directly or indirectly influence the toxicokinetic properties of MeHg has been described in the scientific literature [42]. For this reason, the FFQ collected data on other dietary components, including average weekly intake of: fruit, fresh fruit juice, raw and cooked vegetables, fresh or packaged milk, and yogurt.

### Exposure definition

Fish/whale consumption constituted the source of mercury exposure examined for this analysis. The structure of the FFQ permitted the creation of separate variables representing the usual frequency of consuming each reported species in units of average meals per week in each of the four seasons. We estimated the average number of fish/whale meals/week across seasons. The number of seasons incorporated in the average was determined by each participant’s hair length, with 3 cm corresponding to a single season.

### Outcome definition

The outcome for this analysis was the MeHg concentration measured in hair samples in units of μg/g on a continuous scale. Guidelines generated by Health Canada for interpreting the degree of risk associated with hair-mercury levels provide perspective for interpreting values [43]: hair mercury concentrations ≤6 μg/g are considered acceptable for adult males and females who are not pregnant or breastfeeding [43]; among children under 12 years and women who are pregnant, breastfeeding or of reproductive age, concentrations ≤2 μg/g are considered acceptable [43].

### Statistical analysis

The goal of the statistical analysis was to estimate the association between fish/whale meals per week and hair-MeHg concentration in the study population. We constructed a multivariable linear regression model to estimate beta coefficients and 95% confidence intervals (CIs) as measures of the association between characteristics of interest and hair-MeHg concentration (μg/g). To confirm whether fish/whale consumption frequency could be modeled as a continuous variable, the linearity of the relationship of the continuous form of this variable to hair-MeHg concentration was assessed using a lowess plot (bandwidth: 0.80). Visual inspection of the lowess plots representing the locally weighted regression of MeHg concentration on exposure variables for each season showed that the relationships were not sufficiently linear to justify modeling exposures as continuous variables. Given lack of linearity, the fish/whale consumption variable was converted to a categorical format. A categorical format was the chosen alternative to modeling fish/whale consumption as continuous as a widely accepted approach that results in effect estimates that are easy to interpret, relative to transformations of continuous values. When possible, category boundaries were defined so that there was no more than a two-fold increase in number of servings within a category [40]. The purpose of this was to generate categories within which the effect of interest did not vary substantially [40, 44].

### Variable selection

We used purposeful selection, as proposed by Hosmer and Lemeshow (2000), to identify the best set of adjustment variables [45]. This method follows a change-in-estimate approach, with variable selection decisions based on the extent to which each potential covariate influences the magnitude of exposure effects of interest: All potential covariates were included in a multivariable model and subsequently removed one at a time. If the coefficient of any independent variable changed by ≥10% with the removal of a given covariate, the removed variable was included in the final model [45]. Variables considered for inclusion in the model were: age, sex, community, use of permanent hair treatments, the proportion of consumed fish/whale species harvested from the ocean or local rivers, the proportion of consumed species usually prepared by cooking (versus eaten raw, dried or smoked), and other dietary frequencies, including fruits and vegetables, dairy products or regular use of dietary supplements.

### Bias analysis

Given the potential for MeHg measurement error to produce outcome misclassification, we conducted a quantitative bias analysis using the variation in measured hair-MeHg concentrations of duplicated samples. We calculated the percent change between duplicate analyses of the same participant’s hair. For participants with more than 2 samples analyzed, we used the 2 values that differed the most for the percent change calculation. For participants with more than 2 measurements, the largest difference between measured concentrations was used. To quantify the extent to which measurement error influenced inferences drawn from this analysis, we adjusted the initially measured MeHg values in two ways. First the overall mean percent change and the proportion of the repeated measurements that increased or decreased in value were used to estimate the magnitude of measurement error and frequency of change in either direction in the entire study population. Second, the mean percent change between repeated measurements and the proportion that increased or decreased were stratified by participant characteristics to apply stratum-specific estimates of the magnitude and direction of measurement error to corresponding subsets of participants, selecting at random the participants assigned increasing or decreasing MeHg concentrations. All analyses were repeated using the adjusted MeHg concentrations as outcome variables.

## Results

### Participant characteristics

In the three communities combined, 101 participants provided hair samples and diet data (42 from Aklavik, NT; 32 from Old Crow, YT; and 24 from Fort McPherson, NT). The mean age was 52 years (SD: 15.7; Range: 10–86). Participants were nearly all Indigenous, predominantly identifying as either Gwich’in (60%; 60/101) or Inuvialuit (30%; 30/101). A few participants were of European descent (6%; 6/101) but had been residing in the community for at least 5 years. The study population was disproportionately female (63%; 64/101); none were pregnant or breastfeeding at the time of data collection. Assuming an average growth rate of 1 cm/month, the exposure periods represented in the collected hair samples ranged from approximately 3 weeks to almost 9 years (median: 1.1 year; IQR: 2.1 years).

### Patterns of fish/whale consumption

**Fig. 1:**
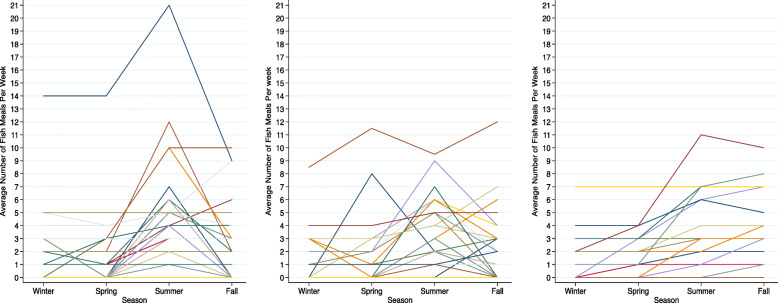
Average number of fish/whale meals per week by season among participants from Aklavik, Northwest Territories (left; n = 45), Old Crow, Yukon (middle; n = 32) and Fort McPherson, Northwest Territories (Right; n = 24), 2016. Each Line represents an individual

Almost all participants (96%; 97/101) reported eating fish/whale in the past 12 months. The data obtained from the fish-focused FFQ was consistent with input from community planning committees, which identified the summer as the season when community members consume fish/whale most frequently (Fig. 1). However, there was considerable variation by species and community (Supplementary File 2, Table 1). The fish species consumed by the largest proportion of participants was Broad Whitefish (*C.nasus*) (83%), followed by Inconnu (*S.nelma*) (42%) and Dolly Varden (*S.malma*) (33%). A large proportion of participants also ate Beluga Whale (*D.leucas*) (42%); 71% (30/42) of those who reported eating Beluga Whale in the past 12 months were from Aklavik, NT, the community with the largest proportion of Inuit residents. Table 2 shows the five most frequent species consumed ≥1 time/week by community. The mean number of different species eaten by participants was 3.5 (SD: 1.9; Range: 0–9). On average, participants reported harvesting most of the species they consumed from local rivers, followed by the ocean and nearby lakes. The distribution of harvesting sites, averaging across species was: rivers, 66.7% (SD: 32.9%; Range: 0–100); the ocean, 21.7% (SD: 27.4; Range: 0–100); and lakes 1.8% (SD: 8.2; Range: 0–50). The proportion purchased from the store, averaging across species, was 2.0% (SD: 7.6; Range: 0–33).

**Table 1:** Five most frequent aquatic species consumed at least once per week by season and community, 101 western Canadian Arctic residents, 2016

Season	Aklavik, NT (*n* = 45)			Old Crow, YT (*n* = 32)			Fort McPherson, NT (*n* = 24)		
	Species	n	%	Species	n	%	Species	n	%
Winter	*S. nelma* *C. nasus* *D. leucas* *S. malma* *C. autumnalis*	9 8 7 5 4	20 18 16 11 9	*C. nasus* *O. tshawytscha* *C. clupeaformis* *O. kisutch* *T. arcticas*	8 6 3 2 2	25 19 9 6 6	*C. nasus* *S. nelma*	9 5	38 21
Spring	*S. nelma* *D. leucas* *C. nasus* *S. malma* *C. autumnalis*	8 8 7 5 4	18 18 16 11 9	*C. nasus* *O. tshawytscha* *C. clupeaformis* *O. kisutch* *T. arcticas*	8 7 4 2 2	25 22 13 6 6	*C. nasus* *S. nelma*	11 6	46 25
Summer	*D. leucas* *S. malma* *C. nasus* *C. autumnalis* *S. nelma*	17 14 14 13 10	38 31 31 29 22	*O. tshawytscha* *C. nasus* *O. keta* *S. aplinus* *O. kisutch* *T. arcticas*	20 9 3 2 2 2	63 28 9 6 6 6	*C. nasus* *S. nelma* *S. aplinus* *D. leucas*	14 10 1 1	58 42 4 4
Fall	*C. nasus* *S. nelma* *D. leucas* *S. malma* *C. autumnalis*	9 8 6 6 4	20 18 13 13 9	*C. nasus* *O. tshawytscha* *O. keta* *L. lota* *C. clupeaformis* *T. arcticas*	9 7 5 4 3 3	28 22 16 13 9 9	*C. nasus* *S. nelma* *L. Lota* *S. aplinus* *S. malma*	15 8 5 1 1	63 33 21 4 4

**Table 2:** Distribution of participant characteristics and stratum-specific mean MeHg concentrations (μg/g) by community, 101 western Canadian Arctic residents, 2016

Participant Characteristics	Total (***n*** = 101)		Aklavik, NT (n = 45)		Old Crow, YT (n = 32)		Fort McPherson, NT (n = 24)	
	n (%)	Mean ± SD	n (%)	Mean ± SD	n (%)	Mean ± SD	n (%)	Mean ± SD
**Demographic Characteristics**								
**Age**								
10–30 years	9 (9)	0.26 ± 0.21	5 (11)	0.17 ± 0.08	4 (12.5)	0.37 ± 0.27	0 (0)	–
31–40 years	15 (15)	0.45 ± 0.60	8 (18)	0.50 ± 0.70	4 (12.5)	0.53 ± 0.66	3 (12.5)	0.20 ± 0.23
41–50 years	14 (14)	0.49 ± 0.34	8 (18)	0.41 ± 0.35	4 (12.5)	0.58 ± 0.35	2 (8)	0.59 ± 0.44
52–60 years	35 (35)	0.79 ± 0.52	14 (31)	0.60 ± 0.35	7 (22)	0.57 ± 0.44	14 (58)	1.09 ± 0.57
61–70 years	17 (17)	0.57 ± 0.31	6 (13)	0.78 ± 0.43	9 (28)	0.47 ± 0.16	2 (8)	0.42 ± 0.00
71–86 years	11 (11)	0.64 ± 0.39	4 (9)	0.42 ± 0.36	4 (12.5)	0.78 ± 0.27	3 (12.5)	0.75 ± 0.56
**Sex**								
Male	37 (37)	0.74 ± 0.51	13 (29)	0.53 ± 0.46	17 (53)	0.68 ± 0.40	7 (29)	1.28 ± 0.49
Female	64 (63)	0.51 ± 0.42	32 (71)	0.50 ± 0.43	15 (47)	0.38 ± 0.20	17 (71)	0.66 ± 0.52
**Hair Treatments**								
No	70 (69)	0.64 ± 0.49	33 (73)	0.48 ± 0.37	20 (62.5)	0.62 ± 0.40	17 (71)	0.95 ± 0.64
Yes	31 (31)	0.51 ± 0.42	12 (27)	0.59 ± 0.59	12 (37.5)	0.4 0 ± 0.21	7 (29)	0.58 ± 0.32
**Fish/Whale Consumption**								
< 1 meal/week	38 (38)	0.42 ± 0.37	18 (40)	0.38 ± 0.34	11 (34)	0.38 ± 0.28	9 (38)	0.55 ± 0.52
1–2 meals/week	21 (21)	0.57 ± 0.48	9 (20)	0.43 ± 0.35	7 (22)	0.42 ± 0.32	5 (21)	1.04 ± 0.62
3–4 meals/week	22 (22)	0.71 ± 0.48	9 (20)	0.57 ± 0.48	9 (28)	0.69 ± 0.38	4 (17)	1.05 ± 0.64
≥ 5 meals/week	20 (20)	0.84 ± 0.50	9 (20)	0.80 ± 0.55	5 (16)	0.77 ± 0.37	6 (25)	0.96 ± 0.58
**Other Dietary Components**								
**Fruit & Vegetables**								
1–3 times/week	20 (20)	0.69 ± 0.52	11 (24)	0.53 ± 0.42	5 (16)	0.48 ± 0.18	4 (17)	1.39 ± 0.49
4–7 times/week	27 (27)	0.61 ± 0.51	12 (27)	0.49 ± 0.39	5 (16)	0.51 ± 0.44	10 (42)	0.81 ± 0.64
> 1 time/day; < 2 times/day	30 (30)	0.69 ± 0.52	13 (29)	0.69 ± 0.52	11 (34)	0.69 ± 0.52	6 (25)	0.69 ± 0.52
≥ 2 times/day	24 (24)	0.59 ± 0.47	9 (20)	0.74 ± 0.63	11 (34)	0.45 ± 0.27	4 (17)	0.64 ± 0.51
**Dairy**								
≤ 1 time/week	29 (29)	0.59 ± 0.47	13 (29)	0.74 ± 0.63	10 (31)	0.45 ± 0.27	6 (25)	0.64 ± 0.51
2–4 times/week	14 (14)	0.61 ± 0.50	9 (20)	0.53 ± 0.40	1 (3)	0.50 ± 0.43	4 (17)	0.96 ± 0.72
5–7 times/week	13 (13)	0.67 ± 0.42	9 (20)	0.59 ± 0.40	2 (6)	0.59 ± 0.00	2 (8)	0.88 ± 0.50
> 1 time/day	45 (45)	0.25 ± 0.16	14 (31)	0.23 ± 0.13	19 (59)	0.37 ± 0.25	12 (50)	0.23 ± 0.24
**Uses Dietary Supplements**								
No	62 (61)	0.67 ± 0.49	33 (73)	0.62 ± 0.56	19 (59)	0.57 ± 0.34	10 (42)	0.86 ± 0.57
Yes	39 (39)	0.58 ± 0.47	12 (27)	0.46 ± 0.36	13 (41)	0.59 ± 0.44	14 (58)	0.94 ± 0.66

### MeHg concentrations

**Fig. 2:**
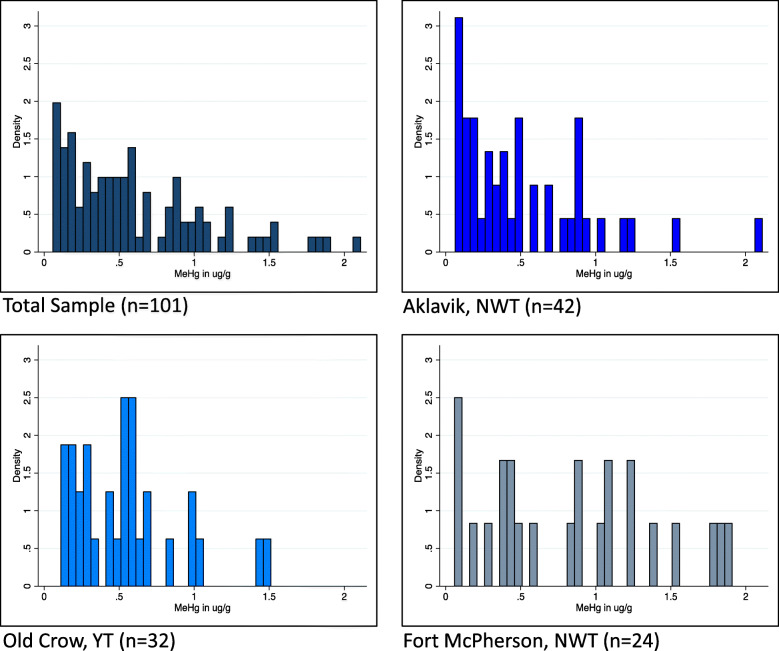
Distribution of MeHg measurements (μg/g) in hair samples among 101 western Canadian Arctic residents by community, 2016

Among participants from all communities combined, the mean concentration of MeHg in hair samples was 0.60 μg/g (SD: 0.47; Range: 0.059–2.07). This varied slightly across communities, with mean values from Aklavik NT, Old Crow YT and Fort McPherson NT of 0.51 μg/g (SD: 0.44; Range: 0.06–2.07), 0.54 μg/g (SD: 0.35; Range: 0.11–1.51) and 0.84 μg/g (SD: 0.58; Range: 0.06–1.90), respectively (Fig. 2). Mean hair mercury levels (μg/g) ± SD stratified by population characteristics are shown in Table 22. No participants had hair mercury levels that exceeded the exposure maximum defined by Health Canada.

### Regression results

**Fig. 3:**
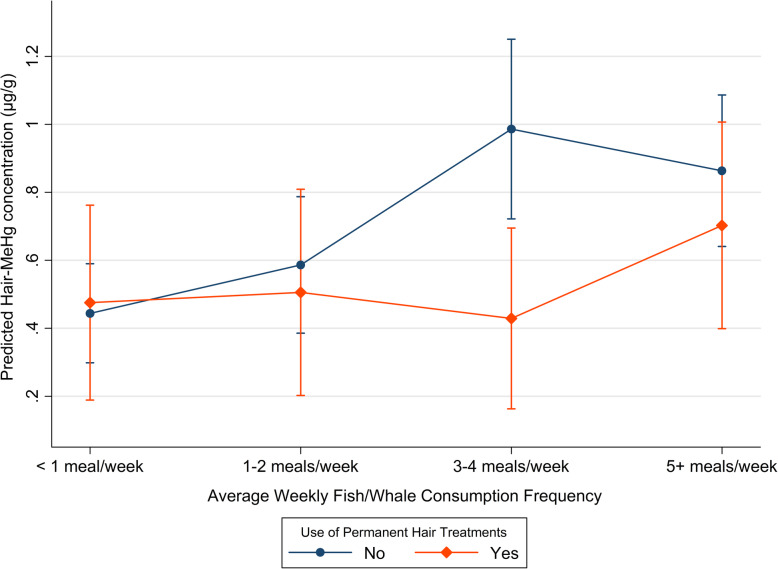
Hair MeHg levels (μg/g) for different categories of fish/whale consumption frequency stratified by use of permanent hair treatments, adjusted for sex, and proportion of fish/whale meals usually prepared by cooking

Because data were insufficient for estimation of species-specific effects on hair mercury levels, this analysis was limited to effects of total fish/whale consumption. Model building procedures yielded the following set of adjustment variables for fish/whale consumption in each season: sex, use of hair dye or other permanent hair treatments, and the proportion of fish/whale meals usually prepared by cooking. There was evidence of statistical interaction between fish/whale consumption and use of permanent hair treatments. Table 3 shows multivariable regression results. Compared to participants who consumed < 1 fish/whale meal/week, hair-MeHg concentration was higher on average among participants who consumed fish/whale more often. A positive dose-response observed in unadjusted beta-coefficients was less striking in the multivariable model, which showed that compared to the referent of < 1 meal/week, the effect of consuming 3–4 meals/week on hair-MeHg concentration was modestly larger than the effect of consuming ≥5 meals/week (Table 3 The confidence intervals of the adjusted effect estimates for the highest 2 levels of fish/whale consumption (3–4 and ≥ 5 meals/week) had lower limits well above the adjusted effect estimate for 1–2 meals/week, however, indicating a clear increase in hair-MeHg concentration among those consuming 3 or more fish/whale meals/week relative to those consuming < 1 meal/week. Assessment of interaction between fish/whale consumption and permanent hair treatment use showed lower hair-MeHg concentrations among participants who reported using permanent hair treatments, relative to those who did not (Fig. 3). Additionally, there was no effect of increasing fish/whale consumption on hair-MeHg concentration for those who used permanent hair treatments and consumed less than 5 meals/week (Fig. 3). Conversely, participants who did not report use of permanent hair treatments had higher hair-MeHg concentrations if they consumed more than 1 fish/meal per week relative to those who consumed < 1 meal/week (Fig. 3).

**Table 3:** Multiple regression of hair MeHg concentrations (μg/g) on fish/whale consumption frequency, 101 western Canadian Arctic residents, 2016

		Unadjusted		Adjusted Φ	
		β	95% CI	β	95% CI
Sex					
Female		*Reference*		*Reference*	
Male		0.227	0.040, 0.414	0.167	−0.014, 0.347
Dye or Perm					
No		*Reference*		*Reference*	
Yes		−0.124	− 0.324, 0.075	0.031	− 0.292, 0.355
Community					
Aklavik, NWT		*Reference*		*Reference*	
Old Crow, YT		0.027	−0.180, 0.234	−0.125	− 0.334, 0.085
Fort McPherson, NWT		0.327	0.101, 0.554	0.249	0.039, 0.459
Proportion Cooked		0.004	0.0001, 0.0075	0.004	- 0.00006, 0.007
Fish/Whale Consumption					
< 1 meal/week		*Reference*		*Reference*	
1–2 meals/week		0.142	−0.088, 0.372	0.142	−0.106, 0.391
3–4 meals/week		0.337	0.092, 0.583	0.542	0.235, 0.849
≥ 5 meals/week		0.422	0.181, 0.663	0.419	0.152, 0.689
Interaction between Fish/Whale Consumption and Use of Permanent Hair Treatments					
Fish/Whale Consumption	**Dye/Perm**				
1–2 meals/week	No			*Reference*	
	Yes			−0.112	−0.607, 0.382
3–4 meals/week	No			*Reference*	
	Yes			−0.588	−1.083, − 0.094
≥ 5 meals/week	No			*Reference*	
	Yes			−0.192	−0.693, 0.309

### Dietary covariates

Few participants reported consuming more than 2 daily servings of fruits and vegetables (Table 2). Conversely, of 101 participants, 45 reported consuming dairy products more than once per day and 40 reported regular use of dietary supplements (vitamins, calcium, fish oil, omega-3 and fibre). Inspection of distributions of hair-MeHg concentrations across categories of dietary components does not reveal clear patterns of association between intake frequencies and hair-MeHg levels. This is consistent with results of the model building procedures, which did not identify the selected dietary factors as important confounders or effect-measure modifiers of the relationship under investigation in the study population. It should be noted, however, that the observed lack of association could reflect insufficient data for precise estimation of effects.

### Bias analysis

Of the 22 samples selected at random in the lab for duplicate MeHg concentration measurement, 4 came from individuals who were among the 10 we selected at random as blind duplicates, yielding 28 participants with duplicate measurements; the median percent change in MeHg concentration between measurements was 14.67% (IQR: 10.75) with 36% (10/28) having second values that were higher than the initial value. The maximum percent change of 159% corresponded to a woman with hair that had been dyed 1 month before sample collection. Excluding this extreme outlier, the mean percent change in MeHg concentration between measurements was 15.8% (SD: 9.95; Range: 3.3–43.8). We performed a bias analysis using two distinct adjustments to hair-MeHg values based on variation in the duplicate measurements. Adjusted MeHg Measurement 1 did not stratify on hair treatment status while Adjusted MeHg Measurement 2 did (see Table 4); means of the adjusted hair-MeHg values were 0.58 μg/g (SD: 0.47; Range: 0.05–2.05) for Adjustment 1 and 0.57 μg/g (SD: 0.50; Range: 0.05–2.87) for Adjustment 2, both slightly lower than the unadjusted mean of 0.60 μg/g (SD: 0.47; Range: 0.059–2.07). Under each of these scenarios, all participants remained at levels below those thought to pose serious health risks. Table 4 compares results from multivariable regression models using the originally measured MeHg concentrations to results of models using values adjusted for measurement error. These comparisons show that laboratory error in measurement of hair-MeHg is not likely to have impacted inferences drawn from this analysis; regardless of measurement adjustments, confidence intervals of effect estimates still indicate that participants at the highest 2 fish/whale consumption levels have clearly higher average hair HeMg concentrations than those at the reference level.

**Table 4:** Sensitivity Analysis: Multivariable regression of hair MeHg concentrations (μg/g) on fish/whale intake, comparing models using measured MeHg values to models using values adjusted for variability in MeHg measurements observed in subsets of the study population, 101 western Canadian Arctic residents, 2016

Fish/Whale Intake (meals/week)	Original MeHg Measurement		Adjusted MeHg Measurement 1 ^a^		Adjusted MeHg Measurement 2^b^	
	β	95% CI	β	95% CI	β	95% CI
< 1	*Ref*		*Ref*		*Ref*	
1–2	0.142	−0.106, 0.391	0.172	−0.129, 0.473	0.112	−0.084, 0.309
3–4	0.542	0.235, 0.849	0.656	0.284, 1.028	0.428	0.185, 0.671
≥ 5	0.419	0.152, 0.689	0.507	0.184, 0.831	0.331	0.120, 0.543

^a^ MeHg concentrations adjusted by the mean percent change of 15.8% estimated among 27 individuals with repeat measurements (excluding 1 outlier). MeHg concentration was increased by 15.8% for a random selection of 36% of participants and decreased by 15.8% for the remaining 64%, based on the distribution of increased or decreased values in the validation subset

^b^MeHg concentrations adjusted according to reported use of permanent hair treatments: Among participants who used hair treatments, 50% were increased by the 38.5% mean percent change observed among 6 participants in the validation subset with reported use of permanent hair treatments; among those who did not use permanent hair treatments, 32% were increased by the 16.2% mean percent change observed among 22 participants in the validation subset who did not use permanent hair treatments

## Discussion

In this study of residents of Western Canadian Arctic communities, hair-MeHg concentration increased with fish/whale consumption, but observed concentrations were all well below levels expected to cause serious health effects according to Canadian government standards [43]. In our analyses, the effect of consuming more fish/whale meals/week was smaller among participants who used permanent hair treatments relative to those who did not; however, data were insufficient for precise estimation of this effect.

Our findings are consistent with the literature pertaining to the relationship between intake of aquatic species and internal dose of mercury as measured in hair [22, 28, 46–60]. Among 66 studies of this relationship identified by systematic review, 44 (67%) showed that increasing fish consumption was associated with increasing hair mercury concentrations [19]. Additionally, our description of the shape of this relationship as nonlinear was supported by the systematic review, which showed that the shape and strength of the relationship varied across populations. As well, our identification of factors associated with the reliability of hair-mercury measurements are consistent with the literature. Specifically, our observation that the use of dyes and other permanent hair treatments were associated with the greatest percent-change between repeated measurements is consistent with evidence suggesting that cuticle damage impacts the retention of compounds and treatment-induced damage can impact strands within the same region of the head to different degrees [61]. It should, however, be noted that our study only had 6 participants who used permanent hair treatments for whom measurements were repeated.

Because there was a wide range of hair lengths among the collected samples (median of estimated growth periods: 1.1 years; IQR: 2.1 years) and hair growth rates vary across individuals, the exposure time windows represented in hair samples cannot be classified perfectly [34, 62]. Although we incorporated hair length in the exposure definition, this may not have adequately controlled for seasonal variations in exposure to MeHg. Since the hair-MeHg measurement was an average concentration across the whole strand, the measured concentration for participants who consume large amounts of fish in only one season and have hair that captures exposure in multiple seasons would not reflect their peak exposure level. However, given all participants had hair-MeHg concentrations that were substantially below the Health Canada cut off for safe MeHg levels, it seems unlikely that any participant would have had excessive exposure at another time of year.

In addition to inaccuracies in classifying exposure time windows based on hair length, the accuracy of reporting food frequencies by season may be higher for seasons closer to the time of data collection. Given that hair length is not associated with fish consumption, however, it seems likely that misclassification resulting from inaccuracies would be independent and non-differential. The accuracy of consumption data and hair MeHg measurements is evident in the consistency of our results with the existing body of evidence on the relation of fish/whale consumption to hair mercury concentration [9, 63], as well as the inverse relationship we observed between hair length and MeHg concentrations. Another source of variation arises from measurements of distal ends of strands that have been growing for extended periods of time; although MeHg remains much more chemically stable in hair relative to other tissues, some of it may be released over time, particularly if the cuticle sustains damage [34, 61, 62]. However, we were unable to collect hair samples in a way that consistently allowed us to identify the root end of the strands, prohibiting us from restricting hair samples to a maximum length. Our analyses were further limited by insufficient data for: precise estimation of effects of individual fish/whale species that participants reported consuming; examining effect modification by dietary factors; or stratifying on permanent hair treatments or hair length.

A major strength of this research its engagement of residents of Indigenous communities in the circumpolar north, who are particularly vulnerable to methylmercury contamination, in strong partnerships with scientists formed to conduct collaborative research that addresses questions for which community members seek answers. Engaging stakeholders in the design and conduct of research is a scientific advancement of increasingly recognized value in translational research. Little health research to date engages people who bear the disease burden in the design and conduct of biomedical research. This study is a novel example of community-driven biomedical research in Indigenous Arctic communities. Community planning committees supported participant recruitment and guided the development of the fish-focused FFQ and the timing of hair sample collection. Community guidance facilitated collection of hair samples following the season of greatest exposure and incorporating commonly used names for aquatic species, which likely improved participants’ ability to provide accurate consumption data.

## Conclusions

This mercury exposure project revealed that a large proportion of western Arctic Canadians regularly consume a wide range of fish species, as well as Beluga Whale. While hair-MeHg concentration increased with fish/whale consumption, hair--MeHg concentrations were well below the level of concern established by the government of Canada in all members of the study population. This study provides reassurance to subsistence fishers in this region, as it suggests that fish/whale consumption does not appear associated with unsafe levels of methylmercury exposure.

## Supplementary information

**Additional file 1: Supplementary File 1.** Fish-Focused Food Frequency Questionnaire developed for this research.

**Additional file 2: Supplementary File 2.** Supplementary Table 1: Fish and marine mammal species consumed at least once in the past 12 months by community, 101 western Canadian Arctic residents, 2016.

## Data Availability

All data collected or created in partnership with Indigenous communities is considered confidential, sensitive and vulnerable to misappropriation. Data generated by this research cannot be shared without explicit permission from the communities who participated in the research. Researchers who are interested in obtaining data can send a proposal to the corresponding author for consideration by community planning committees. Data can only be made available if permission is granted by the community planning committees.

## Abbreviations

MeHg: Methylmercury
CAN*Help*: Canadian North *Helicobacter pylori*
NT: Northwest Territories
YT: Yukon Territory
GC-ICP-MS: Gas Chromatography Inductively Coupled Plasma-Mass Spectrometry
FFQ: Food Frequency Questionnaire
CI: Confidence Intervals
SD: Standard Deviation
IQR: Interquartile Range
