# Supplementary figures and images for: Patterns of fish and whale consumption in relation to methylmercury in hair among residents of Western Canadian Arctic communities
Source: BMC Public Health. 2020 Jul 6;20:1073. doi: 10.1186/s12889-020-09133-2 (PMC7339417; doi:10.1186/s12889-020-09133-2)

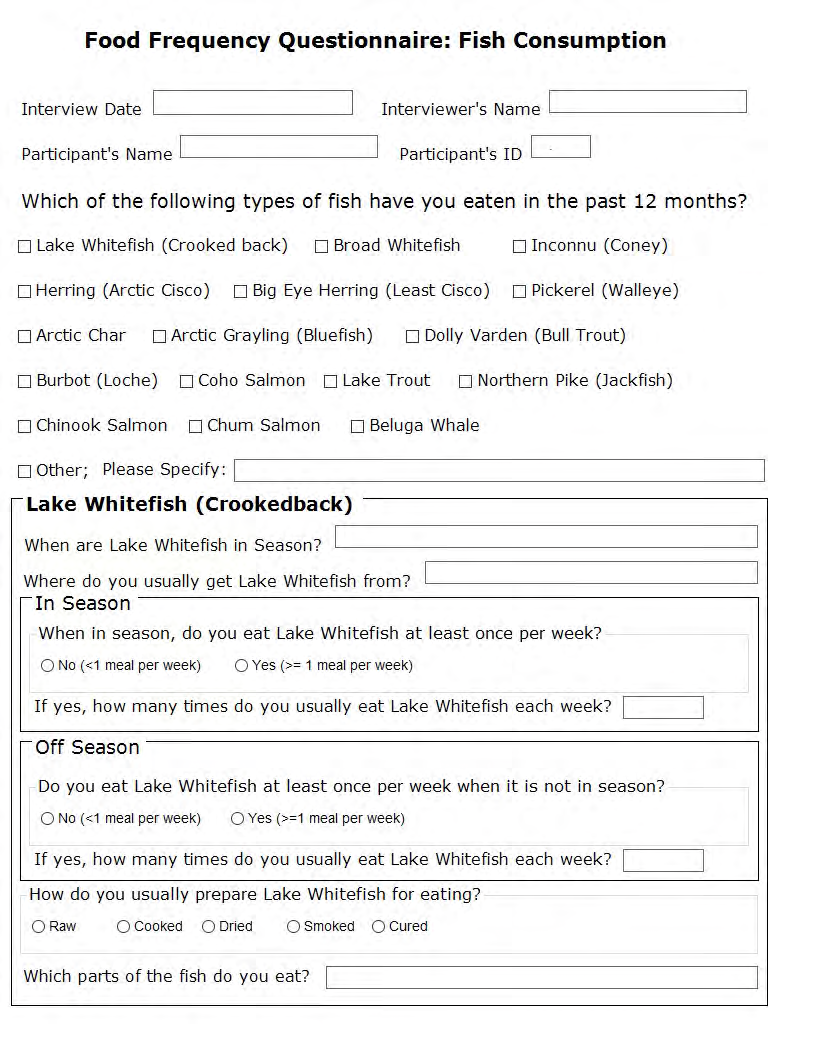


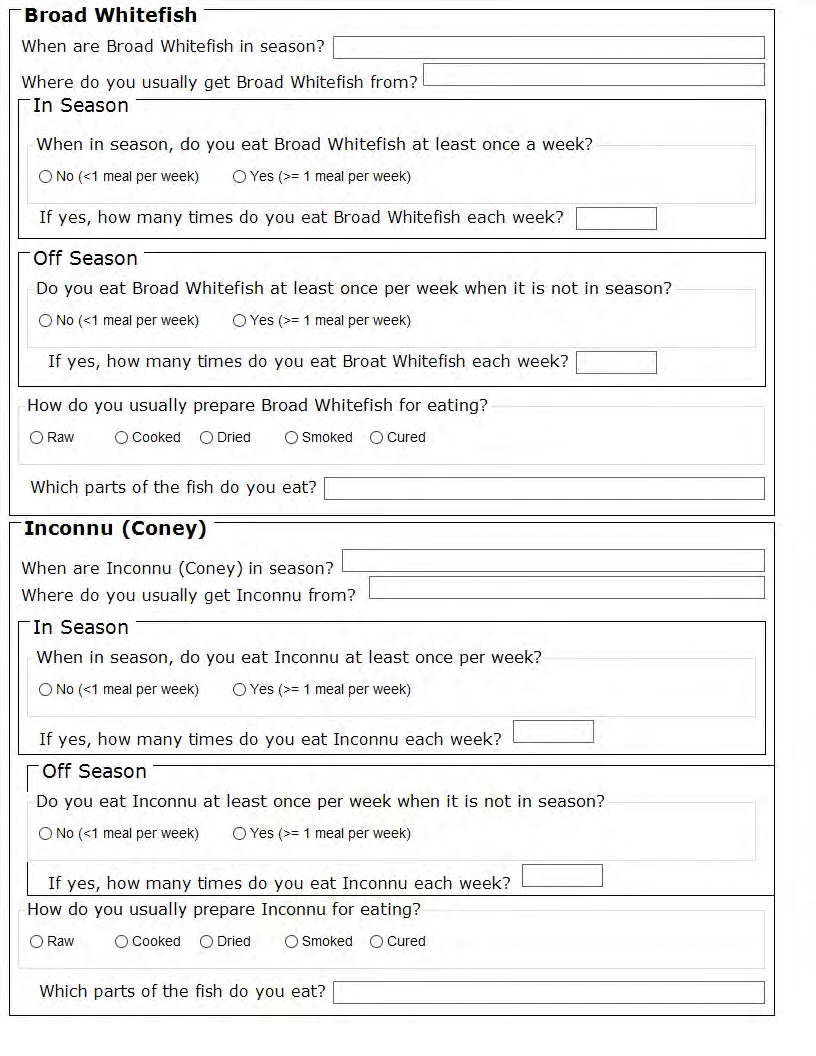


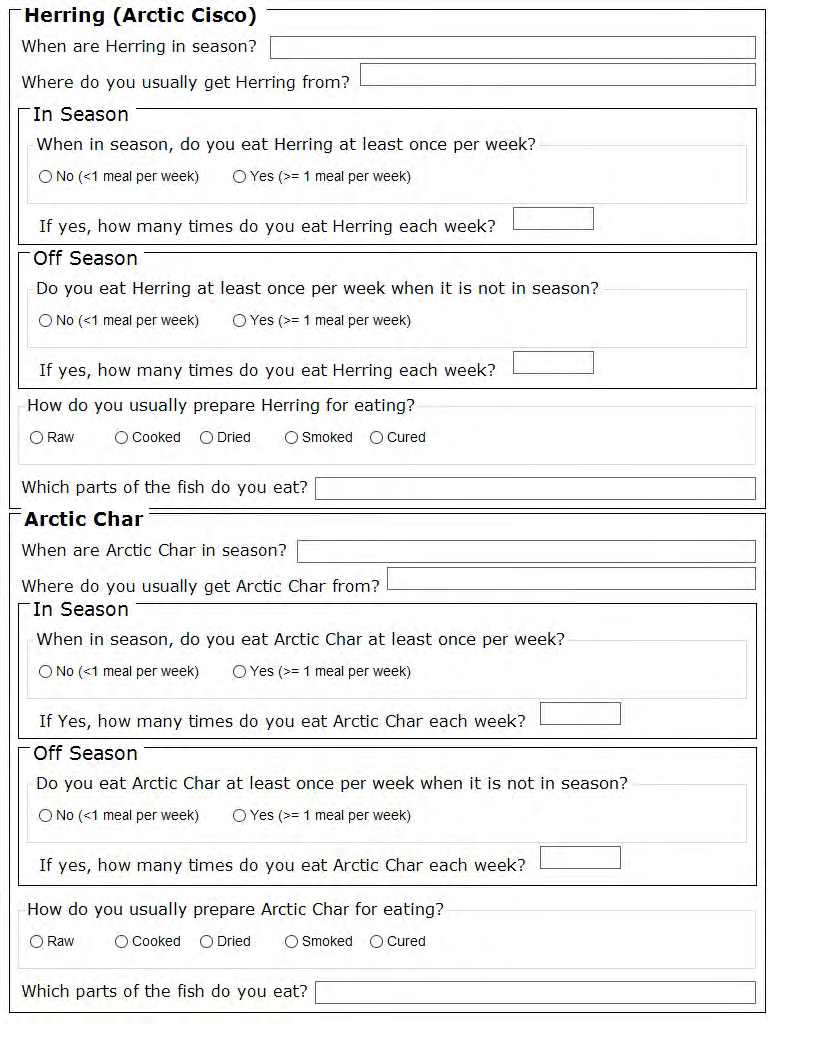


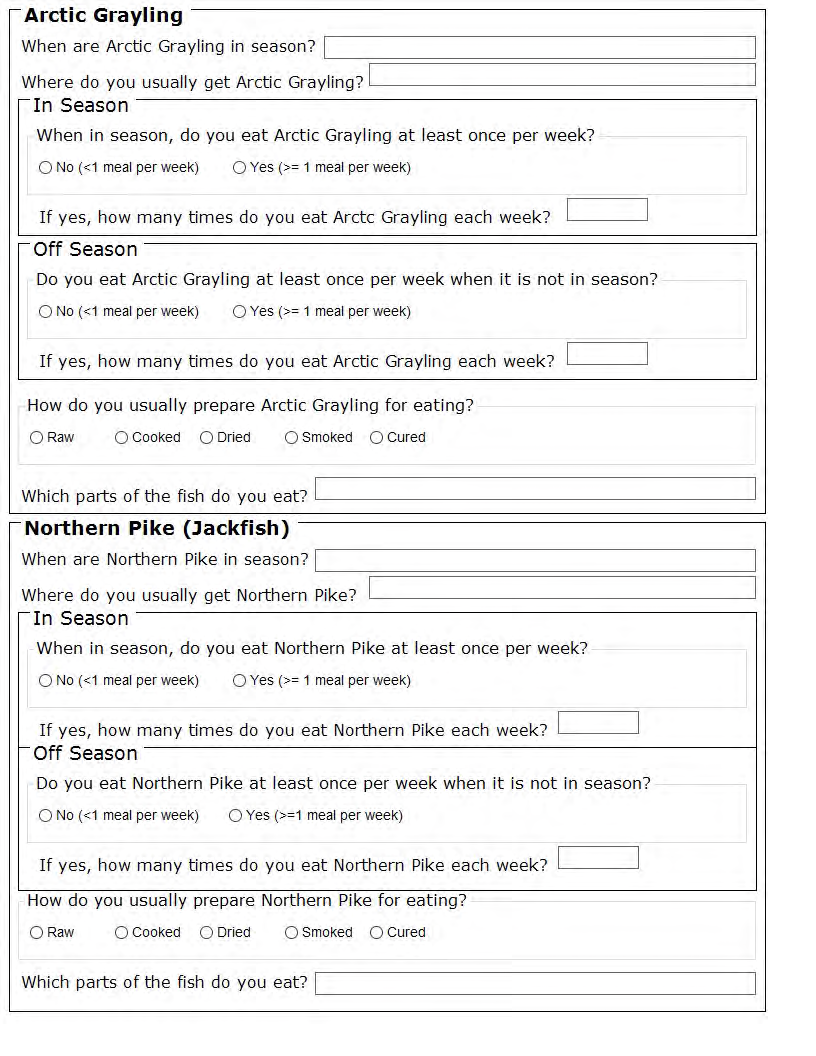


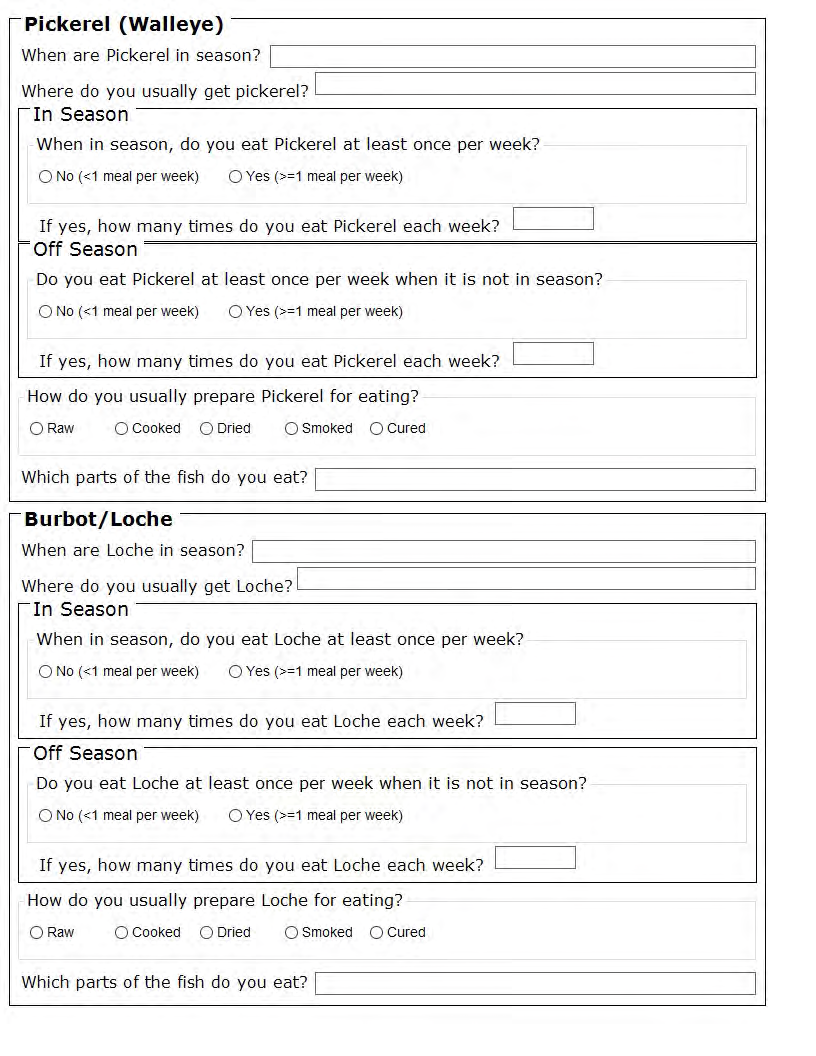


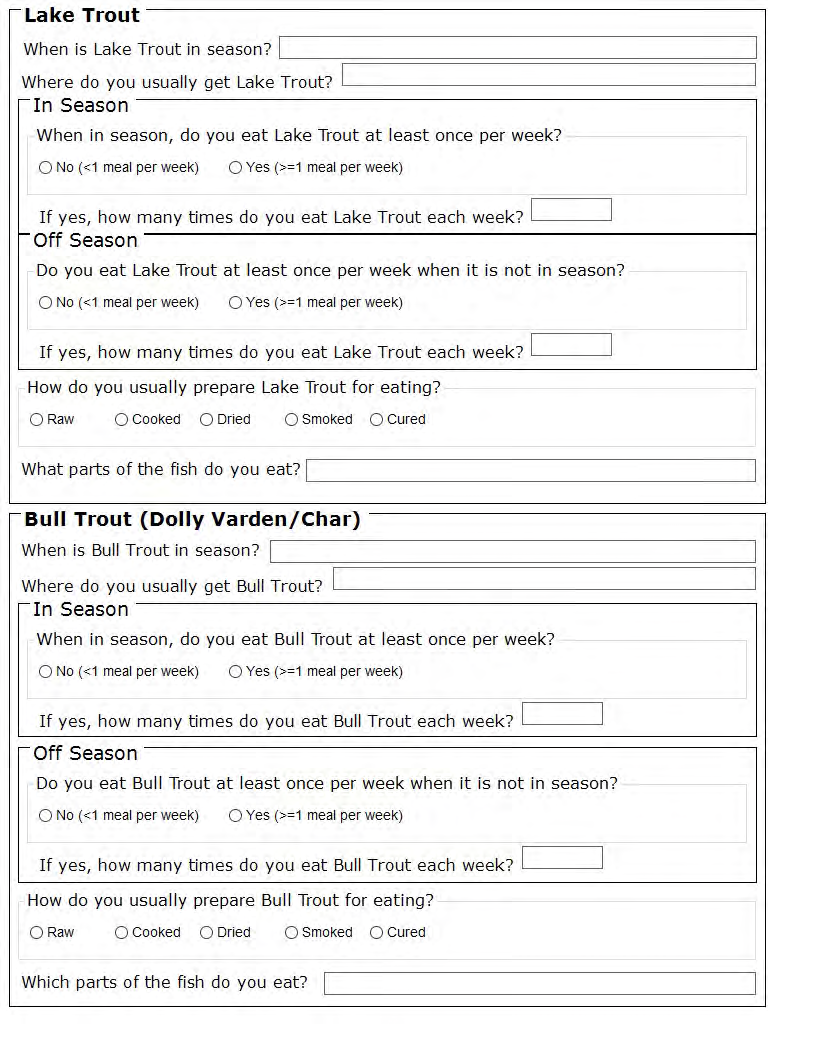


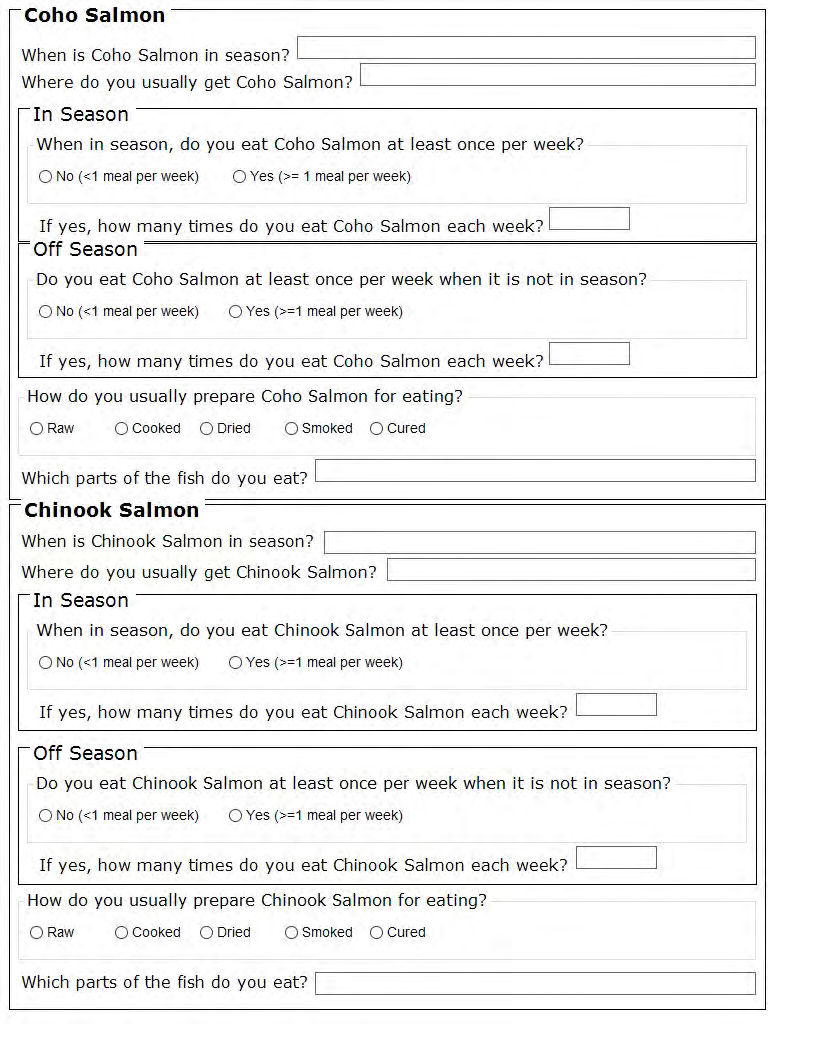


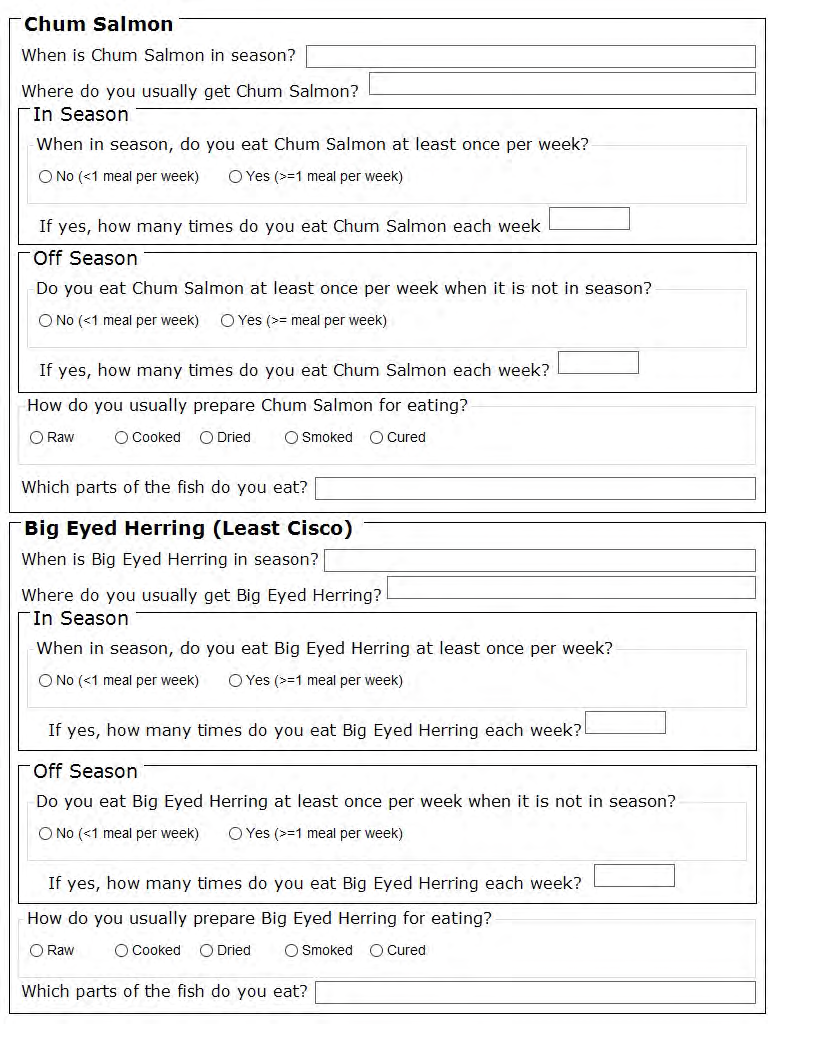


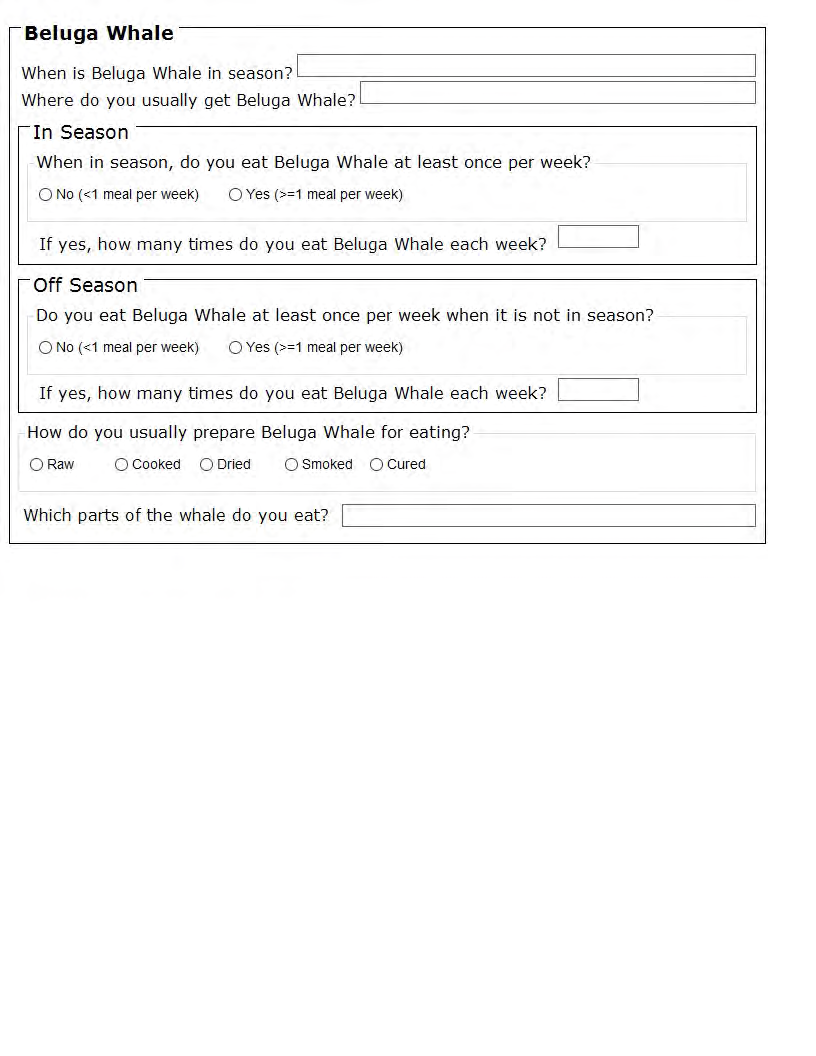

Supplement: Supplementary file 1 — Additional file 1: Supplementary File 1. Fish-Focused Food Frequency Questionnaire developed for this research. [file 12889_2020_9133_MOESM1_ESM.docx]
