# Supplementary material for: Patterns of fish and whale consumption in relation to methylmercury in hair among residents of Western Canadian Arctic communities
Source: BMC Public Health. 2020 Jul 6;20:1073. doi: 10.1186/s12889-020-09133-2 (PMC7339417; doi:10.1186/s12889-020-09133-2)
Supplement: Supplementary file 2 — Additional file 2: Supplementary File 2. Supplementary Table 1: Fish and marine mammal species consumed at least once in the past 12 months by community, 101 western Canadian Arctic residents, 2016. [file 12889_2020_9133_MOESM2_ESM.docx]

**Supplementary Table 1:** Fish and marine mammal species consumed at least once in the past 12 months by community, 101 western Canadian Arctic residents, 2016

| Species | |  | Proportion that Consumed Each Species  in the Past 12 Months | | | | | | | |
| --- | --- | --- | --- | --- | --- | --- | --- | --- | --- | --- |
| Scientific Name | Common Name |  | Aklavik (n=45) | |  | Old Crow (n=32) | |  | Fort McPherson (n=24) | |
|  |  |  | n | % |  | n | % |  | n | % |
| Salmonidae Family | | | | | | | | | | |
| *Salvelinus aplinus* | Arctic Char | | 11 | 24 |  | 3 | 9 |  | 1 | 4 |
| *Salvelinus malma* | Dolly Varden | | 30 | 67 |  | 0 | 0 |  | 3 | 13 |
| *Salvelinus namaycush* | Lake Trout | | 1 | 2 |  | 0 | 0 |  | 5 | 21 |
| *Coregonus nasus* | Broad Whitefish | | 36 | 80 |  | 26 | 81 |  | 22 | 92 |
| *Coregonus clupeaformis* | Lake Whitefish | | 2 | 4 |  | 5 | 16 |  | 0 | 0 |
| *Coregonus autumnalis* | Arctic Cisco | | 18 | 40 |  | 0 | 0 |  | 1 | 4 |
| *Oncorhynchus tshawytscha* | Chinook Salmon | | 6 | 13 |  | 25 | 78 |  | 1 | 4 |
| *Oncorhynchus keta* | Chum Salmon | | 1 | 2 |  | 7 | 22 |  | 3 | 13 |
| *Oncorhynchus kisutch* | Coho Salmon | | 3 | 7 |  | 5 | 16 |  | 0 | 0 |
| *Oncorhynchus nerka* | Sockeye Salmon | | 0 | 0 |  | 4 | 13 |  | 1 | 4 |
| *Oncorhynchus gorbuscha* | Pink Salmon | | 2 | 4 |  | 0 | 0 |  | 2 | 8 |
| *Thymallus arcticas* | Arctic Grayling | | 0 | 0 |  | 9 | 28 |  | 0 | 0 |
| *Stenodus nelma* | Inconnu | | 24 | 53 |  | 1 | 3 |  | 17 | 71 |
| Lotidae Family | | | | | | | | | | |
| *Lota Lota* | Burbot | | 12 | 27 |  | 7 | 22 |  | 10 | 42 |
| Osmeridae Family | | | | | | | | | | |
| *Thaleichthys pacificus* | Eulachon | | 0 | 0 |  | 1 | 3 |  | 0 | 0 |
| Percidae Family | | | | | | | | | | |
| *Sander vitreus* | Walleye | | 1 | 2 |  | 0 | 0 |  | 0 | 0 |
| Monodontidae Family | | | | | | | | | | |
| *Delphinapterus leucas* | Beluga Whale | | 30 | 67 |  | 8 | 25 |  | 4 | 17 |
